# Supplementary material for: Insecticide Resistance of Cimex lectularius L. Populations and the Performance of Selected Neonicotinoid-Pyrethroid Mixture Sprays and an Inorganic Dust
Source: Insects. 2023 Jan 27;14(2):133. doi: 10.3390/insects14020133 (PMC9966739; doi:10.3390/insects14020133)
Supplement: Supplementary file 1 [file insects-14-00133-s001.zip › insects-2158540-supplementary.pdf]

Supplementary Materials

Table S1. Information about field-collected *C. lectularius* populations.

Population	Collection site	Year of Collection
Fort Dix	Ft. Dix, NJ	1973
Aberdeen	Aberdeen, NJ	2018
Bayonne	Bayonne, NJ	2009
Bayonne 2015	Bayonne, NJ	2015
Canfield	Paterson, NJ	2018
Cotton	Paterson, NJ	2018
Dehart	Elizabeth, NJ	2011
Hackensack	Hackensack, NJ	2013
Irvington 624-5G	Irvington, NJ	2013
Irvington	Irvington, NJ	2012
Indy	Indianapolis, IN	2008
Linden 2019	Linden, NJ	2019
Masiello	Paterson, NJ	2016
New Brunswick	New Brunswick, NJ	2021

Table S2. Information about formulated insecticide products

Trade Name	Formulation	Active ingredients	Manufacturer
Transport GHP	Suspension concentrate	22.73% acetamiprid, 27.27% bifenthrin	FMC Corporation, Philadelphia, PA, USA
Temprid SC	Suspension concentrate	21.00% imidacloprid, 10.50% β-cyfluthrin	Bayer Crop Science LP, Research Triangle Park, NC, USA
Tandem	Emulsifiable concentration	11.60% thiamethoxam, 3.50% λ-cyhalothrin	Syngenta Crop Protection, Greensbora, NC, USA
CimeXa	Dust	92.1% amorphous silica	Rockwell Labs Ltd, North Kansas, MO, USA
